# Malaria case management by community health workers in the Central African Republic from 2009–2014: overcoming challenges of access and instability due to conflict

**DOI:** 10.1186/s12936-017-2005-7

**Published:** 2017-09-29

**Authors:** Laura Ruckstuhl, Christian Lengeler, Jean Méthode Moyen, Helle Garro, Richard Allan

**Affiliations:** 1 Swiss Tropical and Public Health Institute, Basel, Switzerland; 2 University of Basel, Basel, Switzerland; 3 The National Malaria Control Programme, Bangui, Central African Republic; 4 The MENTOR Initiative, Crawley, UK

**Keywords:** Malaria, Central African Republic, Conflict, Community health worker, Emergencies

## Abstract

**Background:**

In the Central African Republic (CAR), decades of armed conflict have crippled the public health system. This has left the population without timely access to life-saving services and therefore vulnerable to the numerous consequences of infectious diseases, including malaria. As a response, in 2008 an international non-governmental organization started a network of community health workers (CHWs) in the highly malaria-endemic region of northwest CAR. The area has experienced years of violent clashes between rebel groups and seen hundreds of thousands of people displaced.

**Methods:**

Data from routine patient registers from 80 CHWs working in Paoua and Markounda sub-prefectures were entered and retrospectively reviewed. The time period covered December 2009–April 2014 and hence different stages of conflict and unrest. Several indicators were measured over time, including malaria rapid diagnostic test (RDT) positivity rates, CHW reporting rates, and malnutrition indicators.

**Results:**

Among nearly 200,000 people who consulted a CHW during this period, 81% were found to be positive for malaria parasites by RDT. In total, 98.9% of these positive cases were appropriately treated with artemisinin-based combination therapy (ACT). Only 1.2% of RDT negative cases were incorrectly treated with an ACT. Monthly data from each CHW were regularly reported, with more than 96% of CHWs reporting each month in the first 3 years of the project. However, since the coup d’état in March 2013, the number of CHWs reporting each month decreased as the programme battled the additional constraints of civil war.

**Conclusions:**

Although the political crisis affected the CHWs, the programme showed that it could reach those most vulnerable and continue some level of care at all times. In addition, this programme revealed that surveillance could be maintained in conflict zones. This paper fills a significant gap in the knowledge of malaria control in CAR and this is especially important for agencies which must often decide in a short space of time how to respond effectively to complex emergencies.

## Background

The disruptive impact of armed conflict on a country’s health system has been well documented. It destroys infrastructure, impedes delivery of essential medical supplies, causes large-scale flight of qualified medical staff, and displaces populations; this leads to deterioration in living conditions and increased exposure to infectious diseases [1, 2]. It also leads to decreased access to fundamental health care, resulting in excess morbidity and mortality, which in sub-Saharan Africa are predominantly due to infectious diseases, such as malaria [3].

Geostatistical analysis using a mixed model approach found that the distance from armed conflicts as well as the duration and violence have been shown to have significant influence on malaria parasite rates [4]. This is not surprising when considering that conflict is synonymous with multiple risk factors that are known to increase malaria transmission, including the disruption of control programmes [5]. Unfortunately, evidence today suggests that such emergencies in sub-Saharan Africa are not only increasing but also becoming more complex [6]. Therefore, as conflicts play a crucial role in the operational feasibility of malaria control programmes, it is essential to adapt the strategies used during peace time to emergency conditions and document their implementation [7].

The Central African Republic (CAR) is a country that has been plagued by violence and political instability since its independence from France in 1960 [8]. Frequent and violent clashes between rebel groups regularly force people to flee their homes. The fragile political situation has seen multiple coup d’états since independence [9]. The most recent, in March 2013, saw the country descend into chaos once more, displacing hundreds of thousands of people. The impact on the health system has been dramatic. Essential medicines have been looted from hospitals all over the country, resulting in an inability for the state to provide care [10].

Difficulty to access care combined with a lack of quality data within the health sector in CAR means national health facility-based surveillance is almost non-existent. Such surveillance data are vital to improve knowledge about how the epidemiology of malaria changes as the political, social and population displacement conditions evolve at both local and national level. Data are required to guide future policies and plans adapted to fragile settings.

As a result of this lack of surveillance, very little is known about the epidemiology of malaria in CAR, except reports suggest that it is the leading cause of under-five mortality and accounts for approximately 40% of hospitalizations across the country [11]. In 2008, the international non-governmental organisation (NGO) called The MENTOR Initiative launched a community health worker (CHW) programme in two sub-prefectures of northwest CAR as a way to improve access to malaria case management for vulnerable populations, including children under 5 years, pregnant women and displaced persons. The NGO worked on the assumption that trained CHWs continue to provide basic care to their communities even when these are on the move or in new settlements. The programme began during a time of instability, when clashes between local rebel groups were the main cause of displacement.

Extensive evidence has demonstrated the effectiveness of CHWs for malaria control. As a result, decentralizing medical services from health facilities to the community has become a widely accepted strategy for facilitating early malaria treatment. Studies have shown that CHWs are capable of safely and accurately diagnosing malaria with rapid diagnostic tests (RDTs) if sufficient training and job aids are provided [12, 13]. Furthermore, training CHWs to administer artemisinin-based combination therapy (ACT) to malaria-confirmed patients significantly decreases inappropriate anti-malarial use (provided that ACT supply is maintained). This is an effective way of improving overall child health and reducing patient burden in health facilities [14, 15]. However, these studies have been conducted in politically stable settings, where some form of health system is in place to manage the supply chain and supervise the CHW work. A search of available literature revealed no prior evidence on the impact of a CHW approach in conflict settings.

This study assesses the feasibility and sustainability of a CHW strategy during an ongoing conflict in CAR. The programme in CAR continued running and collecting data from 2009 to 2014, offering a unique opportunity to observe malaria trends during conflict as well as across seasons. It reports on the achievements of the CHW project, its ability to conduct surveillance as well as its challenges and limitations. As the security situation in CAR remains fragile, this analysis is essential to guide the next steps in malaria control in CAR and similar settings and to minimize the consequences of reduced access for those most vulnerable to malaria.

## Methods

### Study site

The CAR is a vast but sparsely populated country of about 623,000 sq km with an estimated population of 4.9 million [16]. It is bordered by Cameroon to the west, Chad to the north, Sudan and Southern Sudan to the east, and the Democratic Republic of Congo (DRC) and the Republic of Congo to the south.

**Fig. 1:**
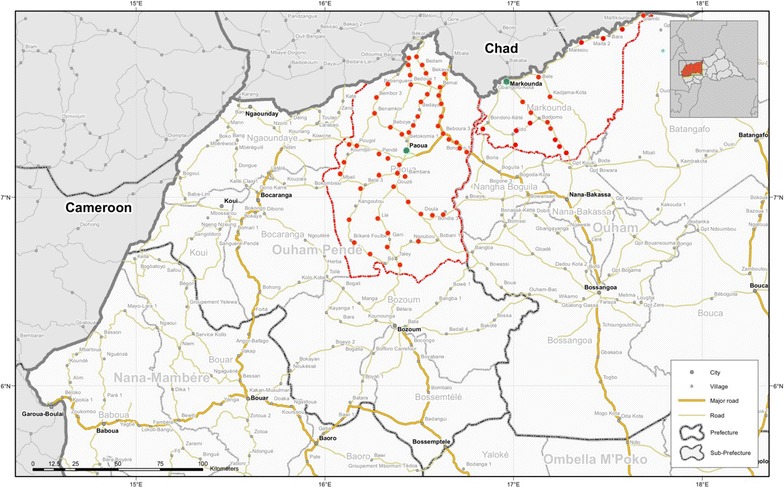
Location of intervention sites in Ouham and Ouham-Pendé sub-Prefectures (*outlined in red*) in northwest CAR. *Each red dot* represents the location of a CHW (Adapted from a UNDP map produced by the Office of the UN Resident and Humanitarian Coordinator 2008)

The intervention and study site is located in Ouham and Ouham-Pendé Prefectures in northwest CAR, where it is estimated that over 200,000 persons are displaced. More specifically, the community-based malaria control programme is located in Paoua sub-prefecture (Ouham-Pendé) and Markounda sub-Prefecture (Ouham) (Fig. 1). The total population in these sub-prefectures is an estimated at 222,000 (200,000 in Paoua and 22,000 in Markounda). While it is difficult to have a clear picture of the number and exact locations of internally displaced persons, many are thought to be in the programme areas. The region is holo-endemic for malaria, with peaks during the rainy season (May–October).

### Description of the intervention

The CHW intervention was designed to target the most vulnerable displaced and host populations with the aim of reducing morbidity and mortality by improving access to quality health care at community level. It began in 2008 in Paoua and in 2011 in Markounda. It is ongoing in both areas. The focus is on malaria case management for children under 5 years and pregnant women, the groups most vulnerable to severe forms of the disease if they are not treated immediately. The programme began with 30 CHWs, known locally as malaria agents, in Paoua and expanded to 60 by 2011, with an additional 20 CHWs that began activities in Markounda in December 2011. In these 80 villages, literate volunteers were selected by the NGO programme management in close collaboration with community leaders. The CHWs were then trained by NGO-employed nurses or physicians over 5 days to implement basic health interventions in their homes, and sensitize their community members on prevention and treatment against infectious diseases. A 3 day refresher training took place every 6 months. Priority of the essential health services was given to malaria diagnosis and treatment. Every patient visiting a CHW from the surrounding area was tested with an RDT (SD Bioline Ag Pf^®^, Standard Diagnostics, Kyonggi, Republic of Korea) and all positive cases were treated with artemether–lumefantrine (AL). For patients with signs of severe malaria, according to the WHO severe malaria management guidelines […], CHWs administered rectal artesunate (Artesiane^®^ Suppogel Artemether 40 mg (Dafra Pharma GmbH, Basel, Switzerland)) before referring them to the nearest health facility. The CHWs also conducted malnutrition screening using the mid-upper arm circumference measurement (MUAC) for all children from 6 to 59 months, classifying them into four categories: well-nourished (>135 mm); at risk of acute malnutrition (125–135 mm); moderate acute malnutrition (110–125 mm); severe acute malnutrition (<110 mm). All children identified as severely malnourished were referred to the nearest NGO-supported treatment centre.

Additional components of the intervention for children under 5 included de-worming with mebendalzole every 6 months, rehydration treatment with oral rehydration solution in case of diarrhoea and administration of vitamin A. For pregnant women, additional interventions included intermittent preventive treatment with sulfadoxine-pyrimethamine, and folic acid supplements, and when available, provision of home delivery kits. Limited prevention campaigns were conducted in this area throughout the study period. In 2010, CHWs distributed 41,194 long-lasting, insecticidal bed nets (LLINs) in Paoua and 4458 in Markounda (totalling approximately one LLIN per household). Additionally, 3830 LLINs were distributed to pregnant women consulting with CHWs in 2010. No LLINs or indoor residual spraying (IRS) campaigns took place in the project area during 2011 and 2012. In the second half of 2013, CHWs distributed 32,900 LLINs in Paoua and Markounda.

All necessary materials and supplies were delivered every month to the CHWs at their homes. Trained staff employed by the NGO supervised each CHW two times per month, enabling quality control and stock replenishment. During these visits, the CHWs received 1 month of anti-malarial stock (based on the consumption from the previous month) plus 2 weeks of buffer stock. During times of increasing insecurity when supervisory visits were sometimes less frequent, the CHWs received 1 full month of buffer stock. To deter falsification of RDT results by CHWs, supervisors cross checked all RDTs during these supervision visits with the number of ACTs given out each month. CHWs were required to pay a financial penalty for any ACTs distributed without a corresponding positive RDT. As additional CHWs were recruited for new villages, the numbers of supervisors also increased proportionally. Each CHW was also responsible for conducting regular information education communication/behaviour change communication sessions on communicable diseases in their villages, as well as being available for specific campaigns, such as distribution of LLINs.

CHWs were responsible for keeping daily registers to record each patient consultation, including basic demographic information, symptoms, test results, and treatment given. The NGO-employed nurses were responsible for collecting the records during their supervision visits and then entering them into a database for basic analysis and reporting. CHWs received a financial stipend for their work, equalling an estimated US$30/month (20,000 CFA) to encourage them to be available for their community without need for additional employment. This amount was decreased from approximately US$50 (30,000 CFA)/month in May 2012 to align with the few Médecins Sans Frontières and International Committee of the Red Cross supported CHWs in other areas of the country.

### Study design and population

To assess the ability of this community-based malaria control programme to provide malaria case management services and surveillance in a crisis setting, a retrospective descriptive study was done based on malaria case management data routinely collected by CHWs between December 2009 and April 2014 (53 months). Whilst the programme was launched in Paoua in 2008, reliable data recording tools were only fully established and implemented in December 2009. Therefore, only data after this date were included in the analysis. The programme in Markounda began activities in December 2011. After April 2014 activities and monthly summary data analysis continued but the detailed data entry of individual patient data stopped due to decrease in funding.

The study population consisted of all suspected malaria cases (defined as patients presenting with fever >37.5 °C or having a history of fever in the last 24 h) as recorded by a CHW as well as pregnant women attending for intermittent preventive treatment. Patients of all ages and from any location were included in the study.

### Data management and analysis

The routinely collected data recorded by CHWs were the basis for this analysis. The CHW registers were collected during monthly supervision sessions and the data were entered into EpiInfo (version 7, CDC, Atlanta, GA, USA) and retrospectively reviewed. Basic clinical and demographic data were recorded (age, gender, village of residence, symptoms, temperature), as well as diagnostic test results, any treatment given and whether the patient was referred to the nearest NGO-supported facility (for example in the case of signs of severe malaria). Data were analysed using STATA 11 (STATA Corp, College Station, TX, USA) to observe trends in malaria incidence during different stages of conflict, to assess the ability of the CHWs to manage uncomplicated malaria cases, and to evaluate the ability of such a programme to continue activities and conduct surveillance during conflict. Annual rainfall data were obtained from The World Bank Group [17].

Malaria incidence rate was calculated as the number of RDT-confirmed cases per month divided by the total population at risk in the area covered by the CHW. Average RDT positivity rates were calculated per CHW instead of exact numbers of cases to take into account the increasing number of CHWs covering a larger area and therefore seeing more patients. The denominator of each monthly calculation also excluded the CHWs that did not report data for that month.

## Results

### Study population

A total of 198,382 people consulted with a trained CHW between December 2009 and April 2014 across both study sites. In total, 68.7% (136,265) were children under 5 years old and 8.9% (17,658) were pregnant women. The ages of patients ranged from newborn to 89 years, and 52.5% were female. In total, 90.3% of these patients presented to CHWs in Paoua sub-prefecture where data were included since December 2009. Only 9.7% presented to CHWs in Markounda which has a much smaller population size and where activities did not begin until December 2011.

### Malaria burden

Each person visiting a CHW was tested for malaria using an RDT. The overall test positivity rate from both study sites was 81.2% (161,052/198,382). Test positivity was higher in children <5 years [83.6% (113,875/136,266)] compared to pregnant women [64.9% (11,463/17,658)] (Table 1).

**Table 1:** Summary of treatment practices for malaria RDT-positive and -negative cases in the total population, as well as for children <5 years and pregnant women

	Total population		Children <5		Pregnant women	
Total tested with RDT	198,382		136,266		17,658	
RDT positive	161,052 (81.2%)		113,875 (83.6%)		11,463 (64.9%)	
RDT positive treated with ACT	159,279 (98.9%)		112,490 (98.8%)		11,372 (99.2%)	
RDT positive treated with rectal artesunate	2378 (1.5%)		2165 (1.9%)		14 (0.1%)	
RDT positive treated with ACT/rectal artesunate	159,695 (99.2%)		112,864 (99.1%)		11,378 (99.3%)	
RDT negative	37,330 (18.8%)		22,391 (16.4%)		6195 (35.1%)	
RDT negative treated with ACT	439 (1.2%)		263 (1.2%)		59 (1.0%)	
RDT negative treated with rectal artesunate	299 (0.8%)		252 (1.1%)		2 (0.03%)	
RDT negative treated with ACT/rectal artesunate	737 (2.0%)		514 (2.3%)		61 (1.0%)	

**Fig. 2:**
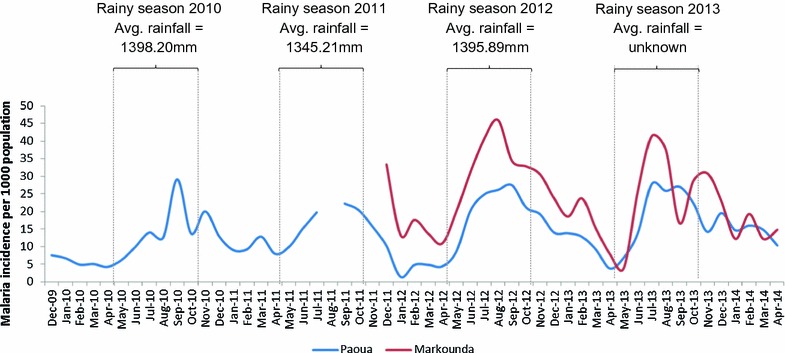
Monthly malaria incidence rate for Paoua and Markounda populations over time. Rainy seasons of each year have been * outlined* with the average annual rainfall where known. Data for August 2011 were lost in the field and this month has been excluded

Malaria incidence rates calculated separately for Paoua and Markounda populations are plotted in Fig. 2. Data collected during the first year of the programme activities in Markounda (which began 4 years after Paoua) saw higher incidences than in Paoua, which decreased over time from an average of 314 per 1000 population per month in 2012 to 273 per 1000 population per month in 2013. Conversely, the annual incidence in Paoua increased from 140 per 1000 population in 2010 to 197 per 1000 population in 2013. There was also a distinct seasonality in the malaria incidence rate at both sites with a peak occurring every year during the rainy season (May–October). Rainfall did not significantly differ between years, although the data are nationwide and not specific to the study sites.

**Fig. 3:**
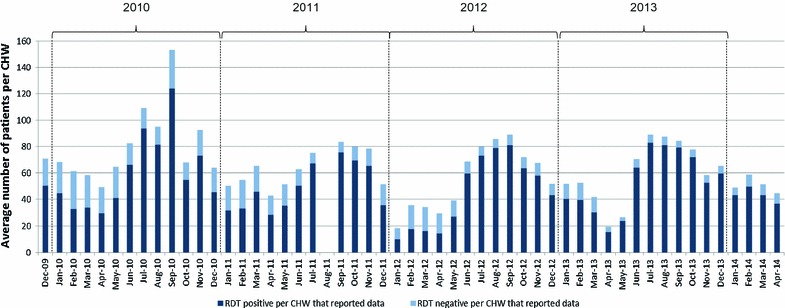
The average number of patient visits per CHW who received a RDT each month according to test result. Data for August 2011 were lost in the field and this month has been excluded

Figure 3 shows the average number of patients consulting with a CHW by their RDT test result. It shows a reduction in the proportion of RDT negative cases over time from 25.6% in 2010 to 11.9% in 2013. It also shows that on average, CHW consulted the most patients per month in the first year of data collection.

### Malaria diagnosis and treatment (with ACT and rectal artesunate)

In total, 99.2% of RDT-positive cases received either ACT or rectal artesunate (Table 1).

Most [98.9% (159,279/161,052)] RDT-positive cases were appropriately treated with an ACT. Furthermore, 416 RDT-positive cases that showed signs of complicated malaria received rectal artesunate instead of ACT before referral to the nearest health facility. There were 1962 RDT-positive cases that received both ACT and rectal artesunate, giving a total of 99.2% of RDT-positive cases that received either ACT or rectal artesunate.

Very few RDT-negative cases [1.2% (439/37,330)] were treated with an ACT. However, 298 RDT-negative cases (0.8%) received rectal artesunate resulting in a total of 2% (n = 737) of RDT negative cases receiving either ACT or rectal artesunate. There was only one RDT negative case that received both ACT and rectal artesunate. Paracetamol was administered to 70.0% (26,125/37,330) of RDT negative cases and 1.3% (2125/158,927) of RDT positive cases.

### Malnutrition indicators for children 6–59 months old

In total 94.1% (127,654/135,595) of children 6–59 months old were tested for malnutrition using MUAC during the data collection period (Table 2). Despite the conflict situation, the majority of children (84.4%) were well nourished. However, 364 children (0.3%) were classified as severely malnourished, 54.5% of whom were female. The proportion of severely malnourished children was not significantly different across the study period, including during the most severe times of conflict. In total, 301 of these severely malnourished children were referred to the nearest health facility.

**Table 2:** Summary of mid-upper arm circumference results for children aged 6–59 months

Malnutrition measurement indicator	Total	
*Children 6–59 months*		
Total measured (mid upper arm circumference)	127,654	
Well-nourished (>135 mm)	114,443 (84.4%)	
At risk of acute malnutrition (125–135 mm)	9508 (7.0%)	
Moderate acute malnutrition (110–125 mm)	3339 (2.5%)	
Severe acute malnutrition (<110 mm)	364 (0.3%)	

### Non-malaria indicators

Of those who had diarrhoea (34,379), 88.0% (30,358) received oral rehydration solution. In total 57,276 doses of mebendazole were given for deworming to children under 5 years, and 704 doses were given to pregnant women. In total, 15,797 people were referred to a health facility for additional follow-up but compliance with this advice to proceed to the nearest health facility was not measured.

### CHW reporting rate

**Fig. 4:**
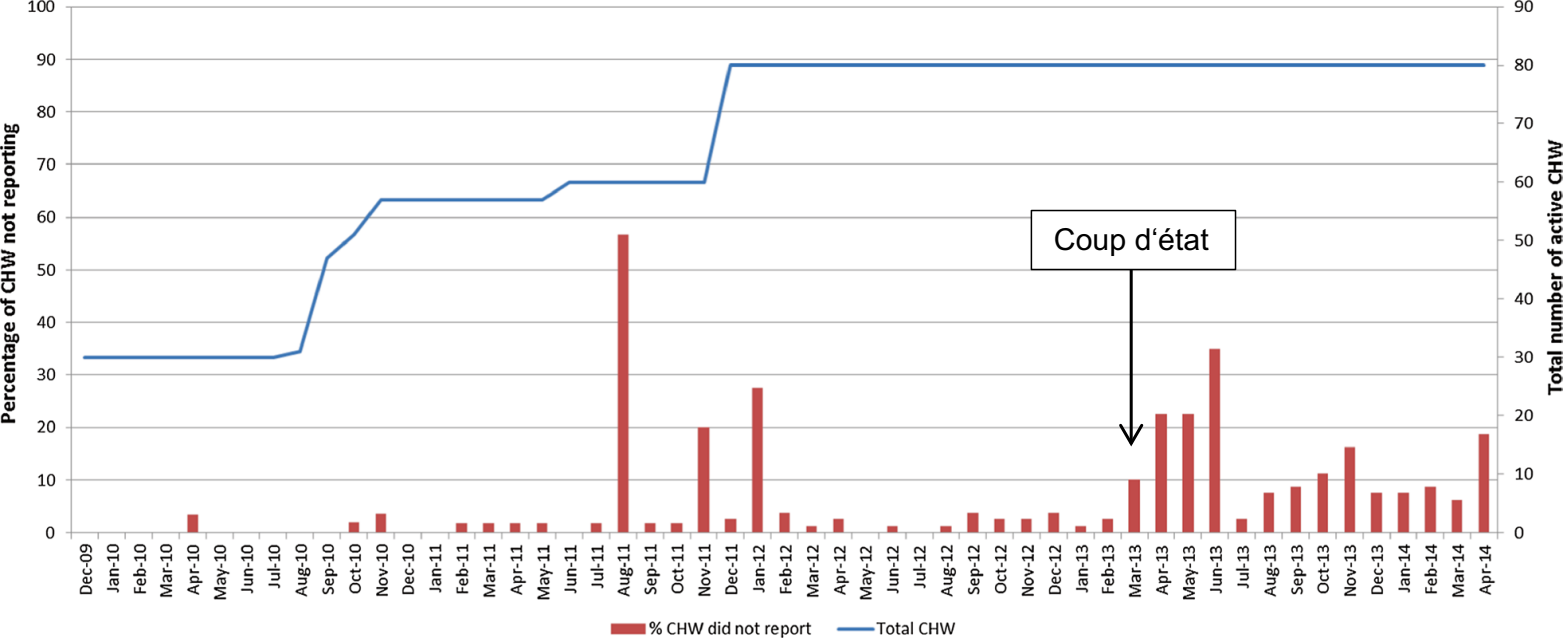
Proportion of CHWs that did not report data, by month. Data for August 2011 were lost in the field and this month has been excluded

During the first year of the study period (2010), 100% of CHWs reported their data each month with the exception of April, October and November when 4% of CHWs did not report. During 2011 and 2012, less than 4% of CHWs consistently did not report their data monthly and in general it was the same CHWs who did not report. Some peaks in non-reporting can be seen in Fig. 4. From the beginning of 2013 when the insecurity increased and political violence peaked, reporting decreased. The most notable increase in non-reporting is seen in the months immediately following March 2013, when the coup d’état occurred.

## Discussion

The CHW programme in northwest CAR plays a pivotal role in ensuring communities have access to effective malaria treatment. It is well targeted to those most vulnerable to severe effects of the disease as 77.6% of those consulting with a CHW were children under five and pregnant women. Analysis of the routinely collected data from this programme highlights that one of its successes was that it has continued, undisrupted, over many years of conflict and instability. The numbers of CHWs even increased over time to access harder-to-reach populations. The programme recruited CHWs from the local population, with the help of the community leaders, which facilitated development of excellent community relations. This in turn led to a deeper understanding of the needs of specific communities. Knowledge of these differences enabled operational adaptations to the intervention to be made over time in order to maximize its effectiveness. For example, the CHW data were used to target IRS campaigns to areas known to experience higher burdens of disease. However, these campaigns took place before the timeframe covered by these data because by the end of 2009 there was a recommendation to shift from conducting IRS towards LLIN distribution. These relationships also meant that the CHW network could be utilized for other activities, including prevention campaigns or special surveys.

The results presented here cover more than 4 years of routine data collection, allowing patterns of malaria seasonality to be monitored while also capturing trends over time during the changing political situations and rebel clashes. Annual malaria incidence rates were extremely high throughout the study period and actually increased over time in Paoua (Fig. 2). This was expected because with little prevention, the programme was managing cases rather than reducing transmission. The assumption when calculating this incidence rate was that the denominator (population at risk in the catchment area of the CHWs) for Paoua and Markounda was constant over time. While the area suffered a high degree of displacement, most is believed to have been local displacement within the same catchment area. People fled into the bush, often for relatively short periods of time, and returned when the situation calmed down. Furthermore, the CHW programme was designed with this in mind so that the CHWs would either flee with the villagers or be able to track people from their community and continue care.

The RDT data showed that there was a decrease in the proportion of RDT-negative cases over time (Fig. 3). This could be because the communities realised with time that the CHW mainly diagnosed and treated malaria. Therefore, community members were maybe less inclined to present themselves to a CHW when they had something that they did not expect to be malaria. This phenomenon was observed when the programme was expanded to a new area in 2014. A high ‘caseload’ was seen in the initial 6 months–1 year when people sought general primary health care for many illnesses, followed by a steady decrease once the limited care capacity of CHWs was understood by the population. Alternatively, when insecurity was higher, people may have waited before seeking care during which time self-limiting viruses causing fever may have resolved. Malnutrition rates did not significantly change over time (trend data not reported) with a low level of severe acute malnutrition diagnosed (0.3%). This level is lower than that measured in southern CAR [18].

The data also showed that during the first and relatively peaceful year of data collection, the CHWs were on average seeing more patients per month than in subsequent years. The most likely explanation for this decrease is that the number of villages with CHWs increased over the years, giving more villages access to their own CHW, therefore sharing the burden of patients. Furthermore, all patients were included in this study even if they travelled from outside the catchment area, something which may have happened more often during more peaceful times. Looking into patient origins and distances travelled would enable this to be explored further. An additional factor which may have contributed to the observed decrease is that CHW motivation may have decreased over time. However, this does not seem likely because even when there was a decrease in the remuneration amount paid in May 2012, the number of patients seen per CHW did not change, suggesting their motivation was not only financial.

Another key strength of this programme was its ability to overcome issues of inaccessibility due to insecurity and extremely poor road conditions, especially during the rainy season. The NGO-supported nurses were able to provide regular extensive training and on-site supervision during which they also maintained the supply chain of essential medicines and materials throughout the periods of highest instability. These factors facilitated the implementation and use of RDTs and led to a very high percentage of RDT-positive cases receiving appropriate ACT treatment (98%). The benefits of intensive supervision and coaching may also explain why such a low proportion of RDT-negative cases were incorrectly treated with ACT (1.2%). This rate is comparable with a recent study in three stable malaria-endemic sub-Saharan African countries that found that CHWs treated 1.03% of RDT-negative cases with ACT [19]. Most cases of incorrect treatment of RDT negative cases with ACT seemed to be by a few individual CHWs. In particular, one CHW treated 111 negative RDT with both ACT and rectal artesunate during the course of 2011. It is unclear why this incorrect treatment practice took so long to be corrected.

The ability to treat such a high proportion of RDT-positive cases is also testimony to the regular stock replenishment with ACT. It is unknown whether there were stock outs of both RDTs and ACT at individual CHW level, but the high treatment rates suggest that this could not have been very often. A drawback of two supervision visits per month and repeated training every 6 months is the expense involved. This brings into question the sustainability of maintaining the quality care without the support given by an international NGO.

One of the main limitations of the programme was the handling of malaria-negative cases. CHWs have restricted options when faced with an RDT-negative patient whose symptoms persist but who does not have the means to travel to a health centre. While the programme made attempts to provide training to recognize the warning signs of other causes of fever, such as respiratory tract infections, dealing with negative cases in the absence of an effective referral and transport system is challenging. It is important to note that this limitation is not just experienced in this CHW programme but is a general problem within the healthcare system in many other sub-Saharan African countries [20]. Even trained nurses in health facilities often default to malaria treatment when another clinical diagnosis can not be made or other treatments are not available. An important next research step to better understand the overall healthcare system would be to follow up referred patients to assess the proportion reaching a referral centre and the quality of care they receive there according to their final diagnosis.

The results presented here show that the overall reporting rate was extremely high. However, this did decrease over time as the project expanded. While the number of supervisors also increased, the larger distances covered could have put an additional strain on their ability to maintain regular supervision visits. This was most evident during the 2013 crisis when the proportion of CHWs not reporting each month increased from less than 5% in February 2013 to over 30% in April 2013. While the project location is almost 500 km from the capital Bangui, the events in the capital triggered a countrywide increase in fragility and attacks and thus the impact was felt in the programme area. During that time, all international staff were evacuated and therefore the flow of money was temporarily slowed as access to banks in Bangui became difficult. This meant that fewer supervision sessions could take place and fewer RDTs and ACT could be delivered. While the initial crisis calmed after several months and relatively normal activities could be resumed, a general insecurity remained. There was very little state authority present in the programme area and NGO teams met increasing difficulties in accessing the communities as a result. This might explain why the proportion of CHWs not reporting remained significantly higher after the crisis (around 10% of CHWs not reporting each month). In spite of the challenges, the timely recording and reporting of data by these CHWs on malaria, nutrition status and diarrhoea was impressive and it was the only efficient surveillance system in the area. The same system has the potential for including other key indicators of diseases found to be affecting the population, e.g., neglected tropical diseases. Through consistent timely analysis and monitoring of these data, the programme was able to continually re-assess and adapt as political and epidemiological conditions evolved.

Data and experience from this study have the potential to serve as an example of effective malaria control intervention in similar environments. In CAR the internally displaced persons are not organized into camps as is often experienced elsewhere, but rather flee into the bush. These displacements, although usually temporary, are frequent and mean that communities are often scattered and consequently very difficult to reach through a conventional health care approach.

This approach has become an integral strategy of the national malaria control programme in CAR since 2012. Unfortunately, implementation has yet to be realised beyond the project area, mainly due to lack of funding. In addition to building this community-based approach into national policies, solid commitment from governments, key international stakeholders and donors must be realised. But ultimately, without peace, health cannot thrive as the State cannot begin to rebuild infrastructure or pay health staff salaries, and trained health staff will not have renewed motivation to return to the most insecure areas. This has been recognized in the Sustainable Development Goal 16, which strives to promote peaceful and inclusive societies to see poverty eradicated and sustainable development thrive which must include health goals [21]. Therefore, the next steps must be to not only continue treating malaria cases and implement prevention, but to also promote peace, without which a sustainable health system cannot be built and the catastrophic impact of the silent yet deadly ongoing epidemic of health system failure will continue.

## Conclusion

Overall, this study demonstrated that decentralizing basic health care to community level during times of instability and unrest through a network of CHWs can be an effective way to manage malaria in hard-to-reach, conflict-affected populations suffering frequent displacements. It showed that supply chains, surveillance systems and continuous capacity building can be maintained in spite of security challenges. However, these humanitarian interventions require extensive resources and do not result in long-term health system development. Peace and good governance are required for strengthening the health care system.
